# Hyperspectral image reconstruction from colored natural flame luminosity imaging in a tri-fuel optical engine

**DOI:** 10.1038/s41598-023-29673-y

**Published:** 2023-02-10

**Authors:** Qiang Cheng, Shervin Karimkashi, Zeeshan Ahmad, Ossi Kaario, Ville Vuorinen, Martti Larmi

**Affiliations:** 1 Department of Mechanical Engineering, Aalto University, 02150 Espoo, Finland; 2 Wärtsilä Finland Oy, 6510 Vaasa, Finland

**Keywords:** Energy science and technology, Engineering

## Abstract

The detection of chemiluminescence from various radicals and molecules in a hydrocarbon flame can provide valuable information on the rate of local heat release, combustion stability, and combustion completeness. In this study, chemiluminescence from the combustion process is detected using a high-speed color camera within the broadband spectrum of visible light. Whereon, a novel hyperspectral reconstruction approach based on the physically plausible spectral reconstruction (PPSR) is employed to reconstruct the spectral chemiluminescence signals from 400 to 700 nm with a resolution of 10 nm to provide 31 different spectral channels. The reconstructed key chemiluminescence signals (e.g., CH*, CH_2_O*, C_2_*, and CO_2_*) from the color images are further analyzed to characterize the chemical kinetics and combustion processes under engine conditions. The spectral chemiluminescence evolution with engine crank angle is identified to comprehend the effect of H_2_ fraction on flame characteristics and combustion kinetics. Additionally, in this study, a detailed kinetic mechanism is adopted to deepen the theoretical understanding and describe the spectral chemiluminescence from H_2_/CH_4_ and H_2_/CH_4_/n-dodecane flames at relevant conditions for various species including OH*, CH*, C_2_*, and CO_2_*. The results indicate that the PPSR is an adequately reliable approach to reconstructing spectral wavelengths based on chemiluminescence signals from the color images, which can potentially provide qualitative information about the evolution of various species during combustion. Here, the reconstructed chemiluminescence images show less than 1% errors compared to the raw images in red, green, and blue channels. Furthermore, the reconstructed chemiluminescence trends of CH*, CH_2_O*, C_2_*, and CO_2_* show a good agreement with the detailed kinetics 0D simulation.

## Introduction

Optical diagnostics have been widely implemented to gain further insight into combustion under engine-like conditions. In general, the optical studies are based on the optically accessible engine and high-speed cameras with or without an external light source (e.g., laser, LED light, etc.) to visualize the ignition and flame behavior in the cylinder^1^. Among all the optical diagnostics techniques, natural flame luminosity (NFL) imaging is the simplest approach to characterize the combustion behavior in the cylinder. NFL refers to the broadband light emitted (e.g., 380–1000 nm or 400–700 nm differ between cameras) by a combustion process during a fired cycle and is used to indicate the locations of the flame chemiluminescence and high-temperature soot particles during mixing-controlled combustion^1^. In NFL imaging, flame luminosity can be recognized by a color camera (CCD/CMOS), which produces an appropriate RGB signal related to the combination of black-body radiation from soot and chemiluminescence from electronically excited reactive species (to be denoted henceforth by “∗”)^2^. Typically, five main electronically excited species formed during chemical reactions are considered for combustion analysis, which are OH* (306.4 nm), CH_2_O* (512–514 nm), CH* (431.5 nm), C_2_* (516.5 nm), and CO_2_* (broadband emission 340 to over 650 nm)^3^. Such radical species are believed to exist in high concentrations near the flame front.

Since the flame is a self-illuminating source, direct chemiluminescence-based analysis can also be conducted by NFL imaging^4^. Therefore, many studies have employed NFL imaging to investigate the combustion characteristics and emissions in optical engines. For instance, Tang et al.^5,6^ applied NFL imaging to analyze the flame front propagation and the tendency of soot formation in reactivity-controlled compression ignition (RCCI) combustion mode. Upatnieks et al.^7,8^ investigated the flame lift-off lengths of low-temperature combustion (LTC) and soot incandescence by using NFL imaging. Vallinayagam Raman et al.^9^ combined high-speed NFL imaging and qualitative fuel-tracer, formaldehyde, and unburned hydrocarbon planar laser-induced fluorescence (PLIF) imaging techniques to quantify the flame probability distribution, the fuel–air mixing, low-and high-temperature heat release (LTHR and HTHR) and UHC distribution characteristics in a partially premixed combustion (PPC) mode. They concluded that the band spectra of species such as C_2_*, HCO*, CH_2_O*, CH*, and continuous spectra of CO* contribute to the blue chemiluminescence in the RGB images, which is likely to be released due to the fuel decomposition processes during the initial stage of the HTHR phase. With the evolution of combustion, the color of the NFL images turns from blue to yellow, which indicated soot formation. Liu et al.^10,11^ employed formaldehyde planar laser-induced fluorescence (CH_2_O-PLIF) and high-speed NFL imaging techniques to detect flame front propagation and auto-ignition in micro-DI RCCI and PPC modes. It was indicated that chemiluminescence often starts from low-temperature combustion due to the relaxation of the excited combustion radicals to their ground states, which represents the start of exothermic chemical reactions and heat release. It should be noted that chemiluminescence exists in the whole combustion process, but it is overwhelmed by strong radiation from luminous flame after soot is generated in the flame^3^. Sreenath et al.^4^ investigated chemiluminescent emission from CO_2_* and pointed toward the potential use of flame chemiluminescence to monitor peak bulk gas temperatures as well as peak heat release rates (HRR) in natural gas engines. For DF or TF combustion, especially under lean conditions, the soot incandescence from the combustion is much lower, which creates a suitable environment for chemiluminescence visualization.

Color-camera-based NFL imaging is a promising tool to visualize the flame characteristics and qualitatively characterize the combustion kinetics. However, the embedded architecture of the digital color camera characterizes only the incident radiation in three discrete bands of signal wavelengths (namely the red, green, and blue channels of the visible spectrum), which makes it difficult to get any information from a specific spectral wavelength^2^. This is because the conventional NFL image post-processing is based on binarization and cannot provide specific spectral information which is crucial for the chemical kinetic analysis. Typically, a spectrometer or a spectrograph is employed to detect the spectral emissions from self-excited species^12^. However, this 1D measurement cannot offer effective spatial–temporal information during combustion. The expensive laser-based measurements such as planar laser-induced fluorescence (PLIF)^13–15^, laser-induced incandescence (LII)^16^, Rayleigh scattering (RS)^17,18^, etc. can provide high-fidelity data for fundamental combustion studies, such as specific species OH, CH_2_O, soot emissions, fuel concentration, and combustion temperature. However, their key issue is related to the high cost and complexity, which limit their wide adoption. Moreover, a single laser can only measure one specific parameter at a time. Therefore, significant opportunities exist for improving the fundamental understanding of combustion characteristics and chemical kinetics based on hyperspectral imaging systems (HISs). While most HISs still rely on either spatial or spectral scanning (via push-broom or filter-wheel principles) to acquire complete hyperspectral images^19^. This inherent limitation of traditional HISs makes them unsuitable for rapid acquisition or acquiring scenes that contain moving objects^20^. To overcome the drawbacks of the HISs, hyperspectral recovery from RGB images becomes an attractive method in high-fidelity multispectral measurements. This method provides the potential to recognize the spectral emissions from the natural flame luminosity imaging. However, reconstruction of the hyperspectral images from an RGB image is a severely ill-posed problem due to (1) their unexpected colorimetric errors after integrating the broadband hyperspectral radiance into RGB values^20^, and (2) the unavailability of the training data for the flame natural luminosity imaging.

From an operation and performance perspective, the RGB colors are physically related to a spectrum: using the spectral sensitivities of an RGB camera, the high-resolution radiance spectra at every pixel in an image can be accurately reproduced from the spectral reconstruction^21^. The advantages of hyperspectral recovery from an RGB system can be categorized as (1) reconstruction of the ground-truth hyperspectral signal based on RGB images providing an opportunity to apply the existing hyperspectral detection/analysis methods instead of the real-time multi-channel capturing, (2) recovering a spectrum that can also reproduce (following the underlying physical conversion and explicit image processing pipeline) the exact ground-truth RGBs^20^, and (3) offering a low cost and possible in high-fidelity multispectral measurements, e.g., turbulent combustion. Unfortunately, the main challenges are (1) the modern spectral reconstruction (SR) solutions ignore the known physical relation between actual spectra and RGBs, and yet give some predictions that contradict the physics, (2) the collecting hyperspectral image datasets are limited in scope (mere number of scenes imaged), (3) low spatial resolution and spectral resolution in the available datasets^17^. Until now, the highest spatial and spectral resolution database is from ICVL (The Interdisciplinary Computational Vision Laboratory, Ben-Gurion University). The images were acquired using a Specim PS Kappa DX4 hyperspectral camera, and a rotary stage for spatial scanning. The raw data was collected at a spatial resolution of 1392 × 1300 and over 519 spectral bands (400–1000 nm at roughly 1.25 nm increments)^21^. However, the current hyperspectral data is mostly used for the natural environment rather than for color-flame calibration. Considering that color photography (electronic sensors or light-sensitive chemicals) records color information by analyzing the spectrum of colors into three channels (red, green, and blue), in natural scenes, it is possible to give rather accurate spectral approximations based on learning approaches^22^.

Motivated by these considerations, we introduce a physically plausible convolutional neural network (CNN) solution to reconstruct the high-resolution hyperspectral images from color NFL images. The advantage of the physically plausible CNN solution is that (1) it can advance both spectral and colorimetric performance of the original network, and (2) it includes color difference-weighted principal component analysis, linear regression with colorimetric correction, and the colorimetrically constrained iterative optimizations and Bayesian inference^23^, which can simulate the ground-truth linear RGBs by calculating the inner products between the measured radiance spectra and the spectral sensitivities of a given RGB camera. However, the application of data augmentation trades off the network performance for model stability against the varying intensity of the flame luminosity. The primary objectives of this work include (1) acquiring, analyzing, and interpreting instantaneous NFL images from an optically accessible engine at a wide range of operating conditions, (2) qualitatively characterizing the NFL features at various conditions, (3) reconstructing hyperspectral images from RGB NFL images to provide deep analysis in species spectral, such as CH_2_O*, CH*, C_2_* and CO_2_* chemiluminescence, (4) evaluating chemical kinetics models based on the chemiluminescence signal. The novelty of this study arises from the reconstruction of the hyperspectral chemiluminescence signal from the color NFL images for detailed flame behavior and combustion kinetics analysis. Furthermore, this study provides valuable experimental data for the validation of the chemical reaction modeling for tri-fuel combustion under practical engine conditions.

The paper is structured as follows.

"Experimental apparatus" section: describes the experimental and modeling approach taken to investigate the combustion characteristics and chemiluminescent species distributions in an optical engine.

"Methodology and image postprocessing" section: introduces the methodology and image postprocessing. Here we thoroughly introduced the physically plausible spectral reconstruction approach for the hyperspectral image reconstruction based on an RGB image.

"Results and discussion" section: the test cases with different H_2_ additions in a pre-mixed CH_4_–Air mixture are applied for NFL intensity, and reconstructed CH_2_O*, CH*, C_2_*, and CO_2_* chemiluminescence analysis. To describe the chemical kinetics mechanism of the chemiluminescent species, the 0D computational model with Cantera is implemented to simulate OH*, CH*, C_2_*, and CO_2_* in CH_4_/H_2_/Air flame and OH*, CH*, CH_2_O* in n-dodecane/CH_4_/H_2_/Air flames.

"Conclusion" section: conclusions on the suitability and advantages of this approach for understanding combustion characteristics and chemical kinetics.

## Experimental apparatus

### Optical engine specifications

**Figure 1:**
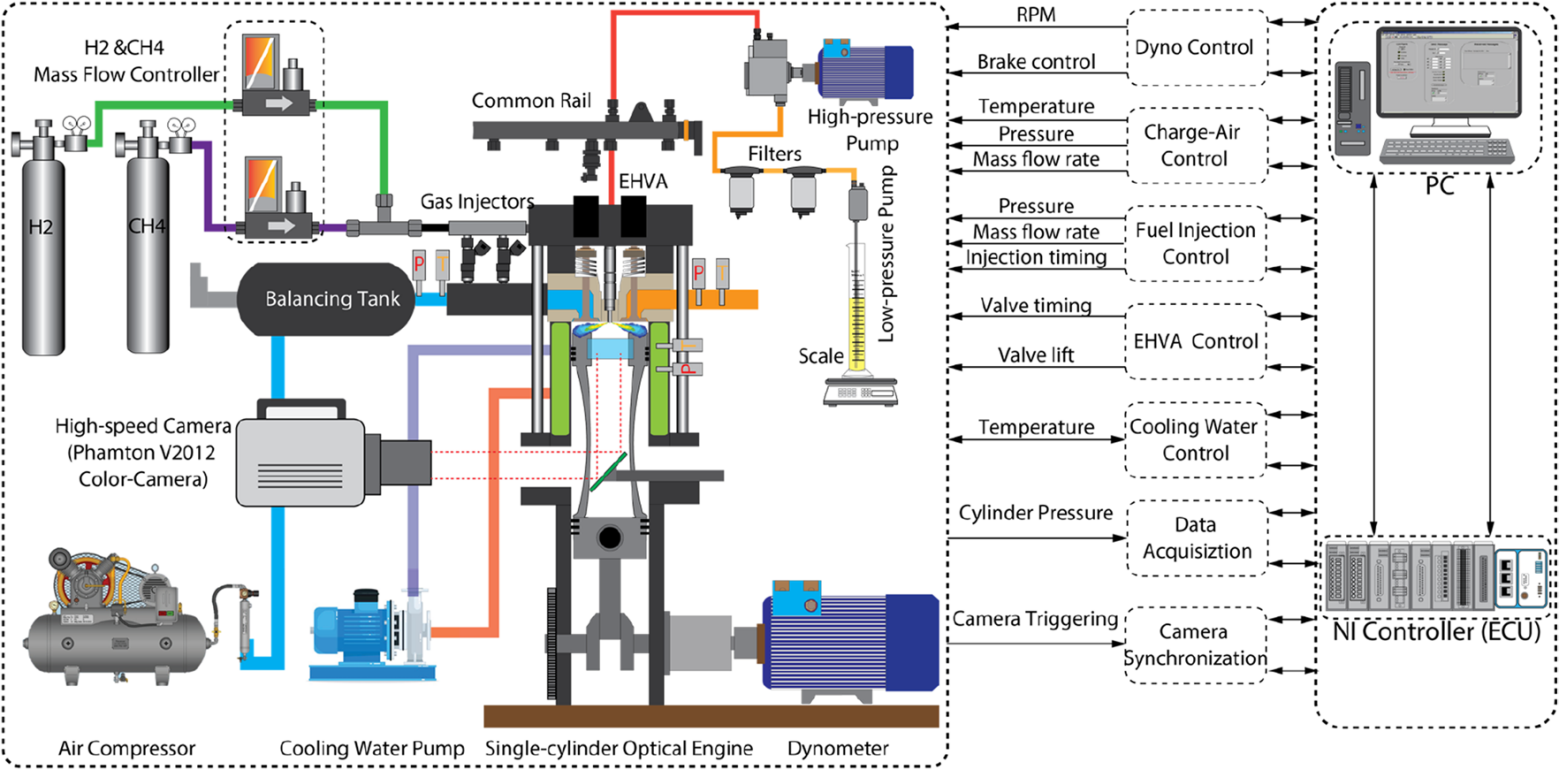
Schematic of the optical engine and high-speed camera location relative to the combustion chamber.

Figure 1 demonstrates the schematic of the optical engine and relevant devices for NFL imaging. The main specifications of the optical engine are listed in Table 1. The single-cylinder optical engine is modified based on an AGCO 84AWI 6-cylinder common rail diesel engine, which can be operated under multi-fuel combustion modes, such as dual-fuel (DF) and tri-fuel (TF). The engine load and speed are controlled by a 45 kW ABB low-voltage motor coupled with a frequency converter (ACS800-11). In this study, the intake manifold is modified to allow the CH_4_-H_2_ mixtures to mix with the air in the intake manifold by using two-port fuel injectors (Bosch NGI2). The mass flow rates of the CH_4_ and H_2_ are separately controlled by two mass flow meters/controllers (EL-FLOW^®^). The valve timing and lift of intake and exhaust valves are precisely controlled by an electrohydraulic valve actuator (EHVA) system. An E-turbo combined with an air mass flow meter (RHM-08 Coriolis mass flow meter, Rheonik Messtechnik GmbH) is integrated to control the charge-air mass flow rate based on a closed-loop PID controller. A 6-hole piezo injector (Bosch CRI 3) is installed at the center of the cylinder head to provide a tiny amount of pilot diesel spray for ignition. A crank-angle encoder is employed to acquire the crank angle signal at a revolution of 0.2°CA. A piezoelectric sensor (type 6125C, Kistler Co., Inc.) with a charge amplifier (type 5011B, Kistler Co., Inc.) at a resolution of 0.2 CAD is implemented to measure the in-cylinder pressure. Before operating the engine, an external water-cooling system is employed to obtain a practical engine condition. The National Instrument field-programmable gate-array (NI-FPGA) and LabView software are employed to monitor, control, and synchronize all signals, such as injection timing and camera synchronization. To allow optical access from the bottom of the extended piston, a sapphire piston window with a view diameter of 65 mm is applied to replace the full-metal combustion bowl. A high-reflection ratio (> 95%) mirror is located at the bottom of the piston window to reflect the combustion signal to a high-speed camera.

**Table 1:** Optical engine specifications.

Parameter	Value
Model	Modified AGCO 84AWI 6-cylinder CI engine
Power	200–298 kW @2100 rpm
Operating speed	1200 rpm
Bore × stroke	111 mm × 145 mm
Length of connecting rod	132 mm
Displacement volume	1402 cm^3^
Combustion bowl	89.9 cm^3^
Geometric compression ratio	16.7:1
Swirl ratio	< 0.1
Diesel injector	Bosch Piezo CRI3 injector
Diesel injector no. of holes × diameter	6 × 90 μm (symmetric)
Diesel injection pressure	1000 bar
Gaseous fuel system	2 × Bosch NGI injectors
Valve system	Electrohydraulic valve actuator

### Optical setup

**Figure 2:**
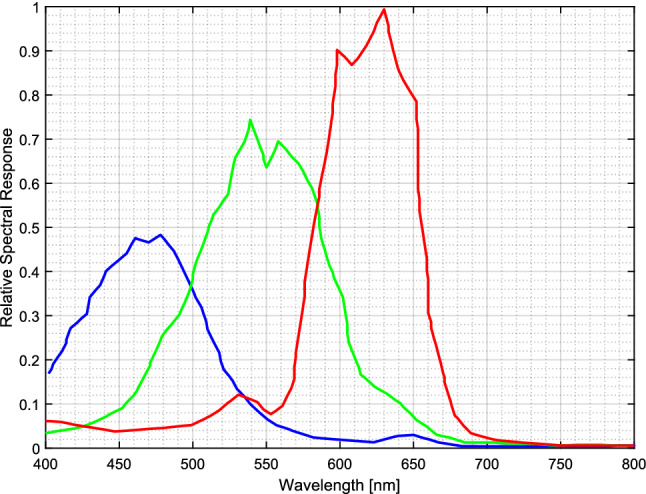
Relative spectral response versus wavelength of Photron SA-Z color camera.

A high-speed color camera (Photron SA-Z) equipped with a Nikon lens (Nikon AF Nikkor 180 mm f/2.8) is employed for NFL imaging with 25 fired cycles. The maximum resolution of 1024 × 1024 pixels with a spatial resolution of 16.4 pixels/mm can be achieved with the exposure of 33.33 μs at a frame rate of 20,000 fps. To observe the spray evolution, a powerful LED ring light (Smart Version Light, RM140) is installed at the front of the camera to illuminate the spray in the cylinder before the start of combustion. The duration of the LED light is 800 µs after triggering. Thus, the temporal resolution of the acquired images was 0.36° CA at an engine speed of 1200 rpm. The color camera has a high sensitivity over a wide visible range, which enables recording the color information of spectral emissions during combustion. The electronic sensors of the camera record the spectrum of colors into three channels, one dominated by red, and the others by green and blue. The spectral response of the color camera describes the sensitivity of the photo-sensor to optical radiation of different wavelengths. In this study, the relative spectral response versus wavelength of Photron SA-Z can be seen in Fig. 2.

### Optical engine operating conditions

Table 2 lists the engine operating conditions. The optical engine is operated at a speed of 1200 rpm with an equivalence ratio of 0.5 (ϕ = 0.5) to estimate the effect of H_2_ addition (from 0 to 60% vol. in CH_4_–H_2_ mixtures) on TF combustion. A closed-loop PID controller is applied to maintain the charge-air mass flow rate of 50 kg/h. The total energy input into the cylinder is ~ 78 MJ/h depending on the ${\chi }_{\text{H}_{2}/\text{CH}_{4}}$, which corresponds to the IMEP of 7 bar. The pilot injection pressure is 1000 bar with an injection duration of 0.174 ms at 7 CAD BTDC, corresponding to ~ 5% energy share ratio (P_ratio_ =  ~ 5%). To protect the engine, the skip fire mode is applied with a skip order of 7 (firing every 7 cycles). For each case, a total of 25 cycles are recorded for data analysis.

**Table 2:** Overview of the engine operating conditions.

Parameter	Unit	Value				
Pilot fuel		EN590				
Start of injection (SOI)	CAD BTDC	7				
$\dot{m}$_air_	kg/h	80				
Equivalence ratio		0.5				
Cooling temperature	^o^C	70				
Pilot energy	MJ/h	13.26				
$\dot{m}$_pilot_	g/h	318.4				
Pilot duration	ms	0.256				
Charge-air temperature	^o^C	25				
H_2_ fraction	Vol.%	0	10	20	40	60
Diesel energy share ratio	%	5				
CH_4_ energy share ratio	%	95	91.9	88.3	79.1	65.4
H_2_ energy share ratio	%	0	3.1	6.7	15.9	29.6
$\dot{m}_{{{\text{H}}_{2} }}$	g/h	0	19.7	42.86	104.05	198.5
$\dot{m}_{{{\text{CH}}_{4} }}$	g/h	1450	1410.7	1364.4	1242	1053.1
H_2_ energy	MJ/h	0	2.36	5.14	12.49	23.82
CH_4_ energy	MJ/h	72.51	70.54	68.22	62.1	52.66
Total energy	MJ/h	76.3	76.7	77.2	78.5	80.5

## Methodology and image postprocessing

### Raw image postprocessing

**Figure 3:**
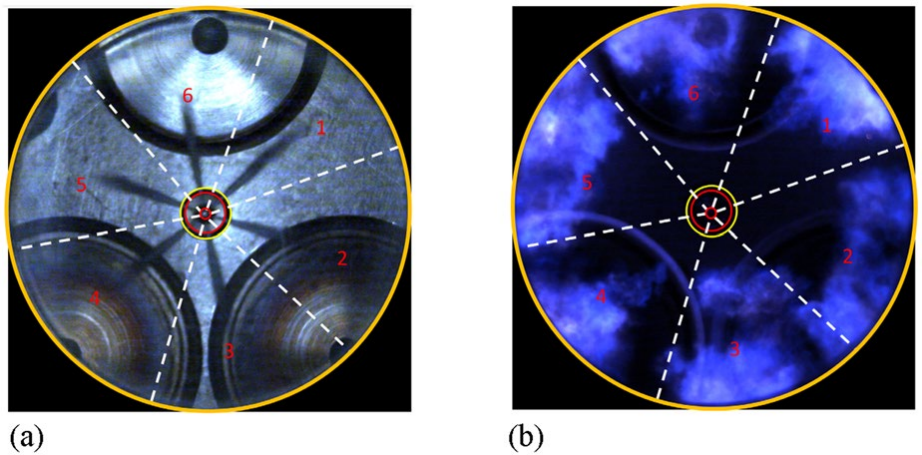
Raw images of the (**a**) pilot diesel spray and (**b**) tri-fuel natural flame luminosity.

Next, we focus on image post-processing. Figure 3 demonstrates the raw images of the pilot spray and the true color of the flames. The ignition first happens in the diesel clouds, where the evaporated pilot fuel is premixed with the hot ambient gas mixture and subsequently starts to react with the available oxygen^24^. Since the pilot fuel is injected from a 6-hole piezo injector, the combustion chamber is divided into six parts based on the spray propagation direction. The blue flame can be observed after the start of combustion due to the CH_2_O*, CH*, CO_2_*, and C_2_* chemiluminescence, which corresponds to the blue and green colors in the visible spectrum. Compared to conventional diesel combustion, in DF or TF combustion, especially under lean conditions, the soot formation is critically inhibited due to the low combustion temperature. Therefore, the high intensity emitted from the soot incandescence can be avoided.

**Figure 4:**
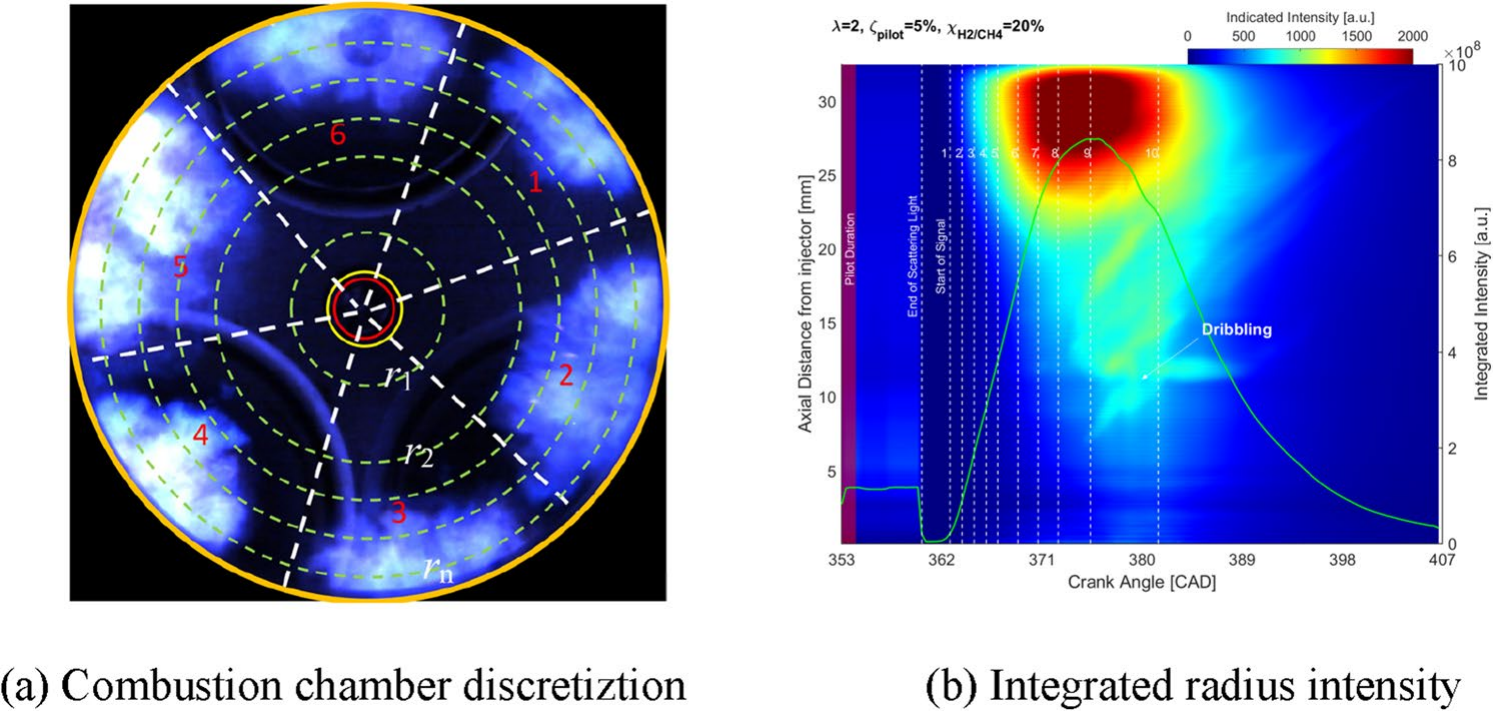
(**a**) Discretization of the combustion chamber radius $\left({r}_{1}, {r}_{2},\ldots ,{r}_{n}\right)$, (**b**) integrated intensity of axial distance from injector tip $I\left({r}_{n}, CAD\right)$ over 25 individual combustion cycles.

Qualitative and quantitative information on the spatial and temporal progress of ignition and the subsequent combustion of pilot-fuel injections into the premixed charge of H_2_-CH_4_-air are analyzed based on the intensity of natural flame luminosity. The intensity of the images is integrated for each axis distance of the injector tip $\left({r}_{1}, {r}_{2}, \ldots ,{r}_{n}\right)$. The spatiotemporal intensity $I\left({r}_{n}, CAD\right)$ plot averaged over 25 individual combustion cycles is constructed by integrating the recorded intensity over a circumference of a circle of discretized radius $\left({r}_{1}, {r}_{2},\ldots ,{r}_{n}\right)$ as presented in Fig. 4a. The intensity distribution of the axial distance from the injector tip (as shown in Fig. 4b) can be defined from the averaged intensity in Eq. (1). The dash white lines with numbers 1–10 in Fig. 4b present the timing of the selected images.

1 $$I\left({r}_{n}, CAD\right)=\sum_{{r}_{n}=0}^{{r}_{n}=r}I\left(x, y, CAD\right)|{x}^{2}+{y}^{2}={r}_{n}^{2}$$

where, ${r}_{n}$ is the discretized radius of the combustion chamber, $r$ is the radius of the view window, CAD is the crank angle degree, $\left(x, y\right)$ are the coordinates on the discretized radius, ${x}^{2}+{y}^{2}={r}_{n}^{2}$. Therefore,$I\left(x, y, CAD\right)$ is the intensity of a pixel at $\left(x, y\right)$ and $I\left({r}_{n}, CAD\right)$ is the integrated intensity on the discretized radius at each crank angle.

### Physically plausible spectral reconstruction (PPSR) based on RGB image

To extract the spectral information from the RGB images, a large number of both hyperspectral and RGB training and validation data is needed to expand the available databases for training/testing. Unfortunately, there is no available training and validation data for hydrocarbon combustion. Recently, only a few databases have become available for hyperspectral-from-RGB reconstruction |(1) ICVL at Ben-Gurion University hyperspectral database (203 images)^21^, (2) Chakrabarti database (77 images)^22^, and (3) the much smaller Yasuma database (32 studio images)^25^. Considering the training size, and spatial and spectral resolutions, the database from ICVL at Ben-Gurion University was selected for spectral image training/testing. The images were acquired using a Specim PS Kappa DX4 hyperspectral camera, and a rotary stage for spatial scanning. The raw data was collected at a spatial resolution of 1392 × 1300 and over 519 spectral bands (400–1000 nm at roughly 1.25 nm increments) in 12 bits^21^. Here, we directly use the .mat file, which has a reduced spectra wavelength from 400 to 700 nm (nm) with 10-nm intervals (31 spectral channels).

Principally, a high-resolution radiance spectrum can be recorded at each pixel in a hyperspectral image. For training the SR model, the inner products between the measured radiance spectra and the spectral sensitivities of a given RGB camera should be calculated to obtain the ground-truth linear RGBs:

2 $${\rho }_{k}=\sum_{\lambda \in\Omega }{s}_{k}\left(\lambda \right)r\left(\lambda \right)$$

where, $k=1, 2, 3$ refer to the red, green, and blue (RGB) channels in a color camera, respectively, ${\rho }_{k}$ denotes the $k$ th channel of camera response, which corresponds to the ground-truth RGB image. ${s}_{k}\left(\lambda \right)$ represents the $k$ th spectral sensitivity function of the camera, which can be seen in Fig. 2. $r\left(\lambda \right)$ is the $k$ th radiance spectral power distribution. λ denotes the wavelength dimension, and Ω is the visible range.

Equation (2) can be vectorized to sample the spectra at a sufficient resolution across the camera spectral sensitivity function:

3 $$\overrightarrow{\rho }={S}^{T}\overrightarrow{r}$$

where $\overrightarrow{\rho }={\left({\rho }_{1}, {\rho }_{2}, {\rho }_{3} \right)}^{\mathrm{T}}$ denotes the RGB vector in three channels, $\overrightarrow{r}$ is the n-dimensional radiance spectra with $n$ to be the number of spectral bands. $S$ = (${S}_{1}$, ${S}_{2}$, ${S}_{3}$) is an $n$ × 3 matrix with its columns to be the spectral sensitivity functions of the RGB camera. Since the databases for training/testing with the spectral range of the camera is 400–700 nm, the spectra are sampled every 10 nm, which means the number of spectral bands is 31, thus $n=31$.

According to the PPSR algorithm, a plausible set concept is defined as the set of all spectra that reproduce a given RGB. The plausible set $\mathcal{L}$ can be defined as:

4 $$\mathcal{L}\left(\rho ;S\right)=\left\{\overrightarrow{r}|{S}^{T}\overrightarrow{r}=\overrightarrow{\rho }\right\}$$

According to Eq. (4), the inner product $\overrightarrow{r}$ can be decomposed into two components, ${\overrightarrow{r}}^{\parallel }\in \mathcal{L}$ and ${\overrightarrow{r}}^{\perp }\in Null\left(\mathcal{L}\right)$. Therefore, $\overrightarrow{r}={\overrightarrow{r}}^{\parallel }+{\overrightarrow{r}}^{\perp }$. Geometrically,${S}^{T}{\overrightarrow{r}}^{\parallel }=\overrightarrow{\rho }$ and ${S}^{T}{\overrightarrow{r}}^{\perp }=0$. Considering an arbitrary $\overrightarrow{r}\in \mathcal{L}\left(\rho ;S\right)$, the ${\overrightarrow{r}}^{\parallel }$ can be derived from subspace projection, written as ${P}^{S}=S{\left({S}^{T}S\right)}^{-1}{S}^{T}$. Then, we obtain ${\overrightarrow{r}}^{\parallel }={P}^{S}\overrightarrow{r}$. Since ${S}^{T}\overrightarrow{r}=\overrightarrow{\rho }$, which gives ${\overrightarrow{r}}^{\parallel }=S{\left({S}^{T}S\right)}^{-1}\overrightarrow{\rho }$. As $S$ and $\overrightarrow{\rho }$ are known factors, then ${\overrightarrow{r}}^{\parallel }$ can be fixed.

To reconstruct the information in ${\overrightarrow{r}}^{\perp }\in Null\left(\mathcal{L}\right)$ (null-space), a basis matrix *N* obtained via singular value decomposition (SVD) on the null-space project matrix ${P}^{N}={I}_{n\times n}-{P}^{S}$. Here, ${I}_{n\times n}$ is the $n\times n$ identity matrix. It should be noted that the rank of ${P}^{N}$ is $n-3$ and the dimension of *N* is $n\times \left(n-3\right)$. Since ${\overrightarrow{r}}^{\perp }\in Null\left(\mathcal{L}\right)$ is the only constraint for ${\overrightarrow{r}}^{\perp }$, the plausible set in null-space and be expressed as:

5 $$\mathcal{L}\left(\rho ;S\right)=S{\left({S}^{T}S\right)}^{-1}\overrightarrow{\rho }\oplus Null\left(\mathcal{L}\right)$$

Based on the calculated null-space basis matrix *N*, the following plausible set can be defined:

$$\mathcal{L}(\rho ;S)=\left\{S{\left({S}^{T}S\right)}^{-1}\overrightarrow{\rho }+N\overrightarrow{\alpha }\left|\overrightarrow{\alpha }\in {\mathbb{R}}^{n-3}\right.\right\}$$

where, $\overrightarrow{\alpha }$ is the null-space coefficients, $\overrightarrow{r}$ and $\overrightarrow{\alpha }$ are one-to-one coupled. Isolating the constrained component ${\overrightarrow{r}}^{\parallel }$ while leaving ${\overrightarrow{r}}^{\perp }$ decided null-space coefficients $\overrightarrow{\alpha }$ can help to search for spectral approximations in an unbounded vector space. This is the key for implementing physically plausible SR based on CNN. More details on reconstructing the null-space coefficients $\overrightarrow{\alpha }$ can be seen in ref.^23^.

**Figure 5:**
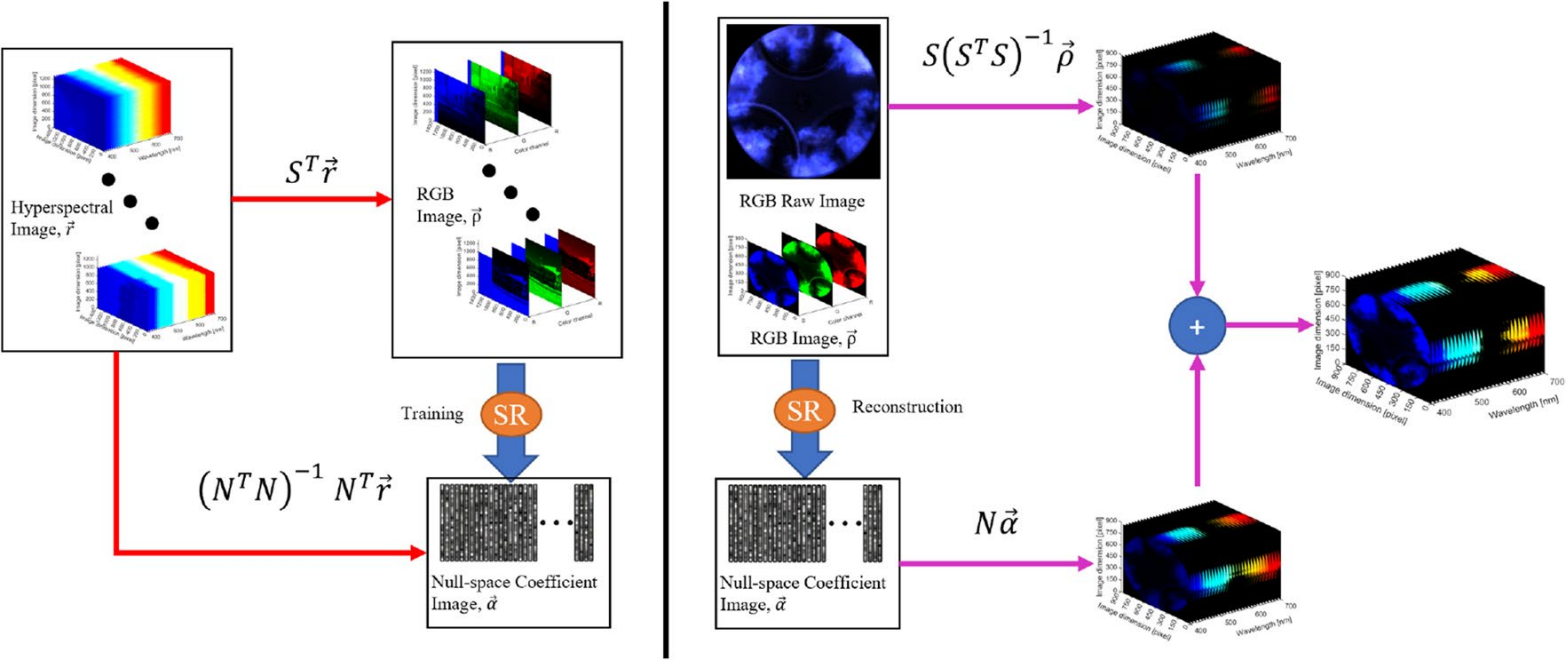
The training (left) and reconstruction scheme (right) of PPSR algorithm.

Figure 5 summarizes the framework of the PPSR. In the training stage (left), hyperspectral images from the databases are trained to map the RGB images to the null-space coefficients images. In the reconstruction stage (right), the camera-subspace projection ${\overrightarrow{r}}^{\parallel }$ is calculated directly from the input RGB $\overrightarrow{\rho }$ while the SR algorithm only concerns the recovery of the null-space coefficients $\overrightarrow{\alpha }$, which subsequently decides the null-space projection ${\overrightarrow{r}}^{\perp }$. As the color reproductions of a spectrum (with the underlying RGB camera’s spectral sensitivities $S$ only depend on their ${\overrightarrow{r}}^{\parallel }$, the reconstructed hyperspectral image is ensured to reproduce exactly the input RGB image^22,23^.

### Image postprocessing and data validation

**Figure 6:**
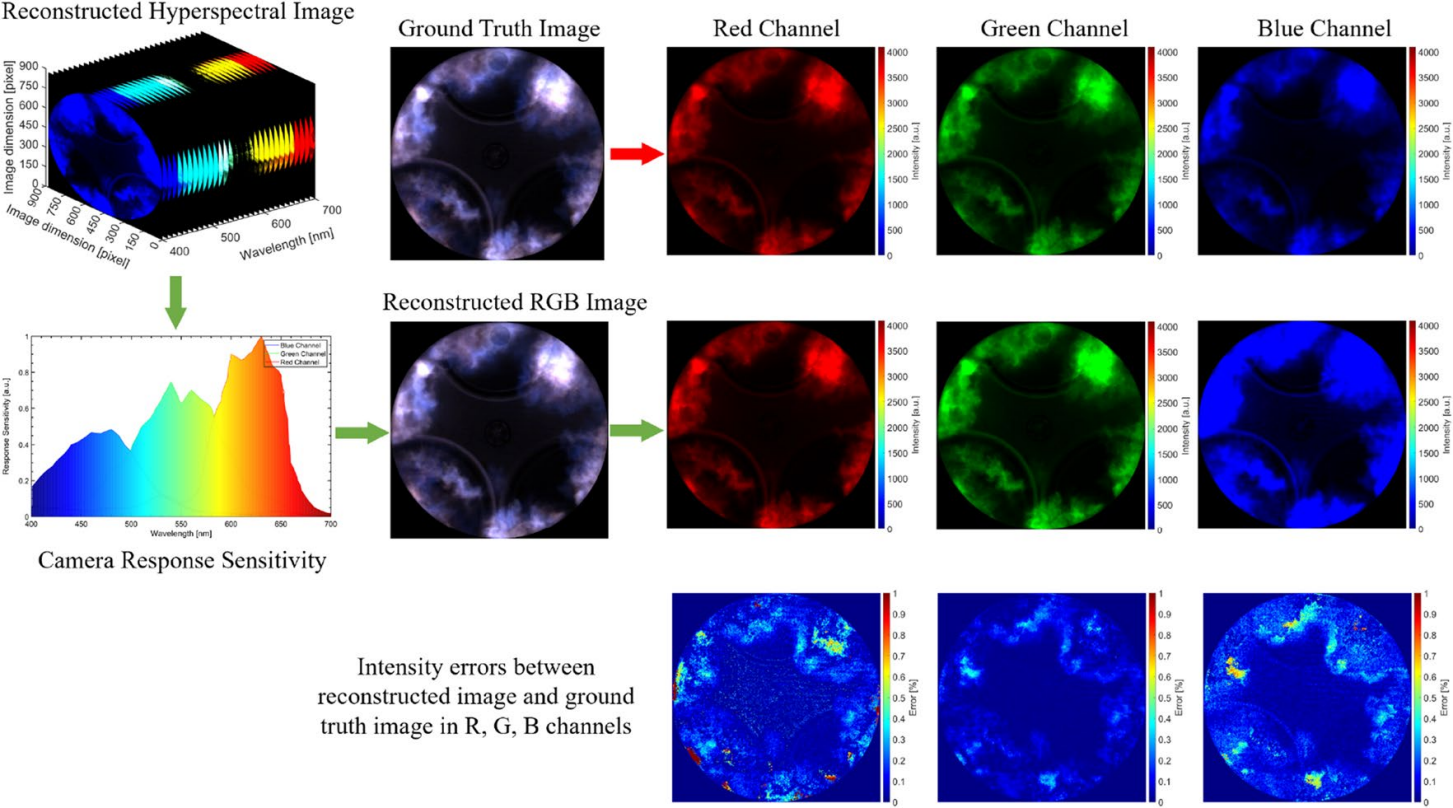
Comparison of the reconstructed RGB image and Raw image.

According to the above explanation, it is known that spectra and RGBs are physically related: an RGB camera integrates spectra with the spectral sensitivities of three different color sensors, resulting in the three R, G, and B channels. To validate the accuracy of the PPSR model, an NFL image with colorful flames is selected to compare the differences between the raw image and the reconstructed ground-truth RGB image, as shown in Fig. 6. The error analysis is based on the R, G and B channels. It is shown that red images have the highest error compared, while the green channel shows the lowest error. However, the overall error of the reconstructed RGB image is less than 1%.

Even though the mathematical validation supports the hypothesis that the PPSR algorithm can accurately reconstruct the spectra image and also can reproduce the exact ground-truth RGB image. However, direct validation is still needed. To provide a direct validation, simultaneous visualization of the NFL and chemiluminescence, including broadband natural flame image (from 400 to 700 nm), CH_2_O* (400 nm), and CH* (430 nm) is performed based on a high-speed color camera (Photron SA-Z) and a bandpass filtered high-speed monochrome camera (Image Doubler + Bandpass filters + Phamton V2012) are captured for validation. More details of the experiments and results can be seen in our previous study^26^.

**Figure 7:**
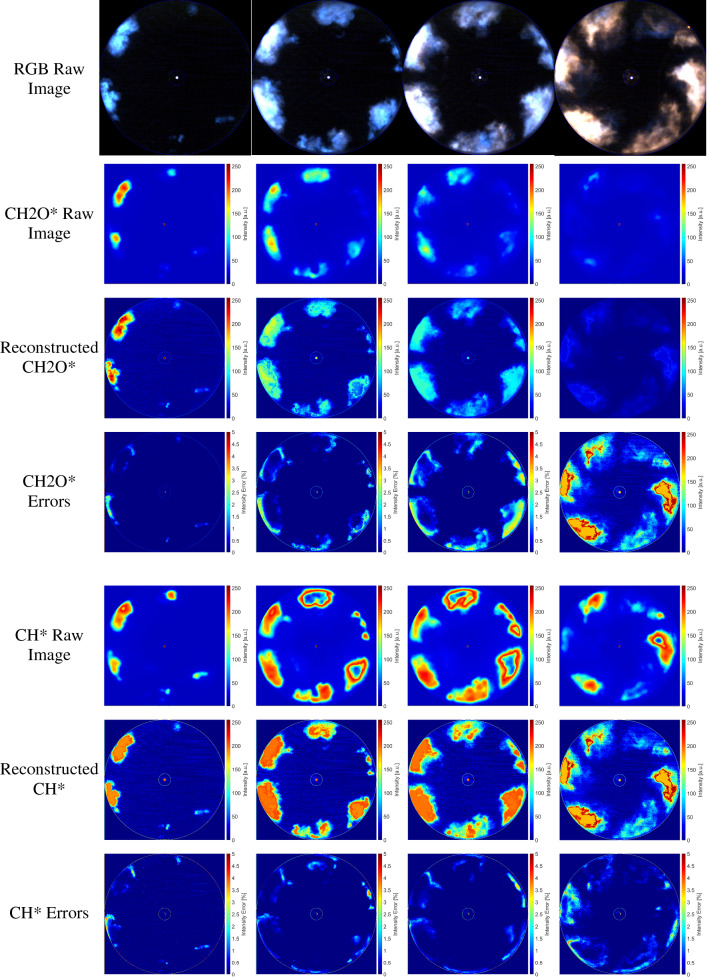
Comparison of reconstructed and raw CH_2_O* and CH* chemiluminescent images.

Figure 7 depicts the comparison of the reconstructed chemiluminescence image (CH_2_O* and CH*) from the NFL RGB image and the raw chemiluminescence image based on bandpass filters. Four images represent the various combustion stages, (1) start of combustion, (2) premixed combustion, (3) the main combustion, and (4) tail combustion in a single cycle are presented for comparison. It can be observed that the reconstructed chemiluminescence images show a similar intensity and species distribution in the whole combustion process. However, since the appearance of saturation in the flame regions in the NFL images after premixed combustion, the reconstructed chemiluminescence signal losses some information. It is also noticed that the reconstructed CH_2_O* and CH* show a higher intensity compared to the raw chemiluminescence signal. This might be related to the differences in the spectral sensitivities of the color camera and monochrome camera, as well as the different transmission and reflection ratios of the beam splitter and image doubler which were used to obtain colorful NFL image and chemiluminescence images (e.g., CH_2_O* and CH*) simultaneously. It can be seen that the reconstructed chemiluminescence images and raw images show a similar feature at various combustion stages. The maximum errors between reconstructed and raw CH_2_O* and CH* chemiluminescence are less than 10%, as shown in Fig. 7. The maximum error mostly takes place at the tail combustion stage due to overexposure and image saturation.

**Figure 8:**
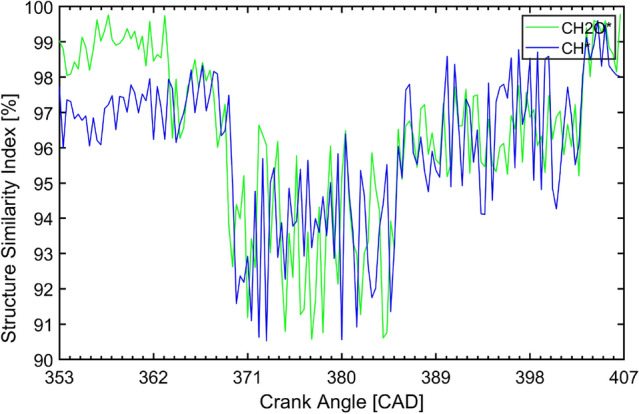
The structure similarity index of the CH_2_O* and CH* between the raw image and reconstructed image.

To further estimate the accuracy of the reconstructed hyperspectral image. A structure similarity index method (SSIM) is employed to assess the quality of the reconstructed image^27,28^. The structure similarity index of the CH_2_O* and CH* between the raw image and reconstructed image can be seen in Fig. 8. The structure similarity index versus the crank angle indicated that the reconstructed CH_2_O* and CH* image provides high-quality data for the specific wavelength (or self-excited species). Before the initiation of the combustion, it shows very high similarity due to the specific illumination from the LED light. After the reaction starts, the structure similarity index immediately reduces to a relatively lower level. The further intense combustion reaction occurs around 371–380 CAD, which shows the lowest similarity index due to the saturation of the local high-temperature reaction zones. During tail combustion, the similarity index increases again due to the disappearance of the saturation in the image.

## Results and discussion

In this section, the combustion characteristics and flame features of tri-fuel combustion in an optical engine will be comprehensively analyzed based on NFL and PPSR algorithms under various ${\chi }_{\text{H}_{2}/\text{CH}_{4}}$ conditions.

### NFL imaging-based analysis

**Figure 9:**
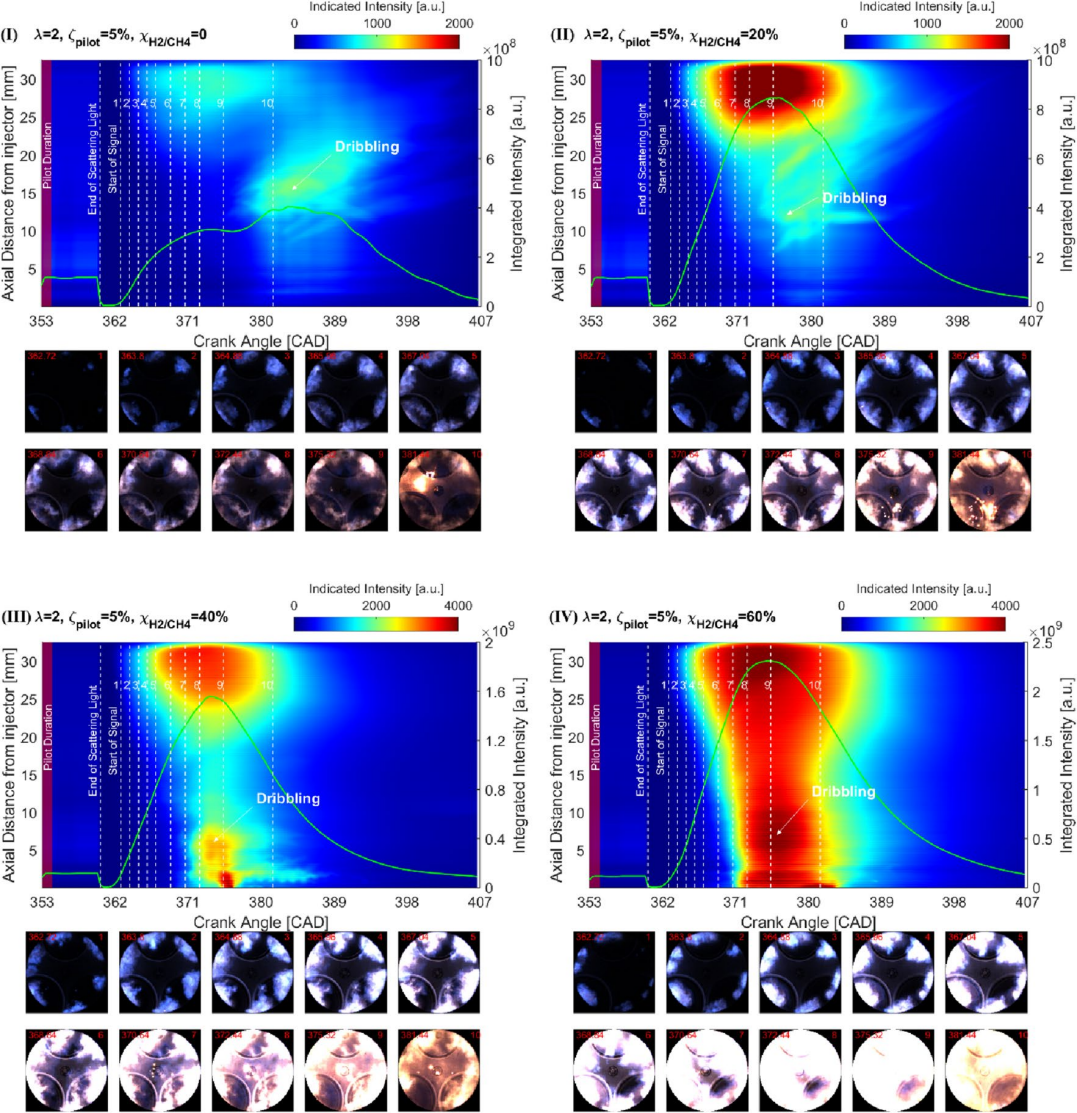
The true-color natural flame luminosity (bottom) and axial intensity distribution (upper) of TF combustion with different H_2_ concentrations, (**I**) ${\chi }_{\text{H}_{2}/\text{CH}_{4}}$ = 0, (**II**) ${\chi }_{\text{H}_{2}/\text{CH}_{4}}$ = 20%, (**III**) ${\chi }_{\text{H}_{2}/\text{CH}_{4}}$ = 40%, (**IV**) ${\chi }_{\text{H}_{2}/\text{CH}_{4}}$ = 60%.

Figure 9 presents the true-color images and axial intensity distributions of TF combustion at various H_2_ concentrations (${\chi }_{\text{H}_{2}/\text{CH}_{4}}$ = 0%, 20%, 40%, and 60%). A total of 10 images from the start of the signal to CA90 (the crank angle at 90% of the cumulative heat release) are selected for comparison. The green profile presents the integrated intensity image versus the crank angle.

The appearance of the flame can be used to define the start of the combustion or IDT. During the start of combustion, the flame that takes place close to the bowl edge shows a blue color due to the band spectra of species such as chemiluminescence of OH*, HCO*, CH_2_O*, and CH* relatively have short spectral wavelengths (< 500 nm), and continuous spectra of CO_2_*, which also contributes to the short wavelength emissions^8^. These species are likely to be released due to the fuel decomposition process during the initial stage of the high-temperature heat release (HTHR) phase^8^. With the evolution of combustion, the flames are getting brighter and the color turns from blue to yellow due to the consumption of the low-temperature species (e.g., HCO*, CH_2_O*, CH*) and the increase of the CO_2_* and H_2_O vapor, as well as the soot formation. It should be noted that bright spots in the vicinity of the injector tip are a consequence of injection dribbling.

Increasing the ${\chi }_{\text{H}_{2}/\text{CH}_{4}}$ leads to more intense combustion, which shows brighter flames with the engulfing area. It can be seen that the flames propagate based on ignition kernels are quenched due to the lean condition ${\chi }_{\text{H}_{2}/\text{CH}_{4}}<40\%$. With increasing the ${\chi }_{\text{H}_{2}/\text{CH}_{4}}$, the flames propagate towards the center of the chamber, due to the border flammability limits and higher burning velocity of the H_2_. According to the observation, the flame features of TF combustion in the optical engine can be categorized as three stages: (1) dark blue at the start of the combustion, (2) bright blue during the main combustion, and (3) bright orange in the tail combustion. The explanation is related to the following reasons: (1) at the beginning of ignition, the temperature in the cylinder is relatively low, and the main spectral emissions of the combustion are OH*, HCO*, CH_2_O*, and CH*, which emit short spectral wavelengths, (2) during the main combustion, the spontaneous reaction in the flame front produces high-temperature, which enhances the reaction sequential with the high natural flame intensity, (3) with the depleting of the pre-mixed mixture, the more difficultly combustible dribbling droplets start to burn and format soot accompany with the residual gaseous, which emit bright orange light. The results also indicate that the addition of the H_2_ extends the lean limits for the charge-air mixture, which may lead to more complete combustion and less unburn CH_4_ and hydrocarbons.

### Hyperspectral reconstruction from color NFL imaging

The hyperspectral reconstruction based on the PPSR algorithm is implemented to gain further insight into the flame spectral emissions under various H_2_ concentrations in the CH_4_–Air mixture. The distribution of the spectral wavelength versus the crank angle, and the key chemiluminescence emitters such as CH_2_O*, CH*, C_2_*, and CO_2_* are reconstructed from the NFL images.

**Figure 10:**
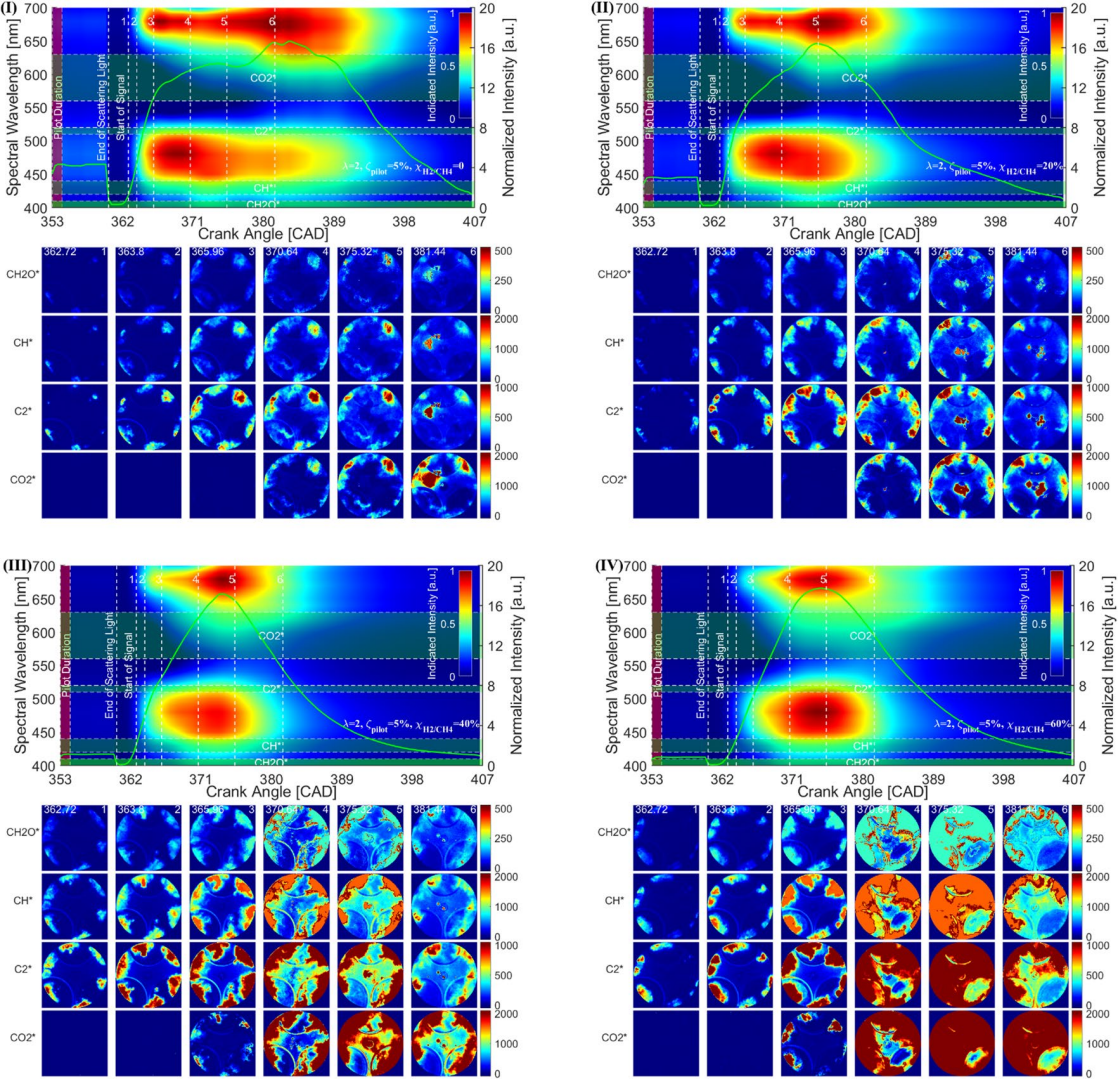
Hyperspectral reconstruction from color NFL imaging for characteristics of TF combustion under various ${\chi }_{\text{H}_{2}/\text{CH}_{4}}$ conditions.

Figure 10I–IV demonstrates historical spectral wavelength and reconstructed chemiluminescence images versus crank angle under various H_2_ concentrations in the CH_4_–Air mixture. Color contours in each subplot (top) summarize the spectral-temporal evolution of the indicated spectral intensity. The green line in the top subplot shows the normalized intensity. The locations of the interested chemiluminescence emissions are marked in a transparent dash box. False-color images represent the chemiluminescence signal reconstructed from RGB NFL images. Four self-excited species such as CH_2_O*, CH*, C_2_*, and CO_2_* are extracted from the reconstructed hyperspectral images applied to characterize the combustion process. Since the available databases have a low spectral resolution in the context of training/testing, the increment for the spectral wavelength is kept at 10 nm for each chemiluminescent radical. For instance, the hyperspectral signal is reconstructed for CH_2_O* between 400–410 nm, CH* between 420–440 nm, C_2_* between 510–520 nm, and CO_2_* between 560–630 nm. The criteria of the selection are to avoid overlap of the spectral wavelength with each radical.

The evolution of the spectra indicates that two bands of broadband luminosity with a band extending from 400 to 520 nm and a band extending from 580 nm towards longer wavelengths can be observed in TF combustion. Where the first band is attributed to CH*, CHO* CH_2_O*, and C_2_* chemiluminescence, and the second band is related to the overlapping H_2_O vibration–rotation bands 580–740 nm and the CO_2_* 340–650 nm. Increasing the H_2_ concentration equally increases the intensity emits from the first band but relatively reduces the intensity emits from the later band.

The reconstructed chemiluminescence emissions of CH_2_O*, CH*, C_2_* and CO_2_* under various ${\chi }_{\text{H}_{2}/\text{CH}_{4}}$ can be seen in Fig. 9 (bottom). CH_2_O*, CH*, C_2_*, and CO_2_* chemiluminescence have been reported as HRR indicators^29–31^, location of the reaction zone^32,33^, an indicator of the equivalence ratio (ER) of the reacting mixture^34,35^. As observed in the reconstructed chemiluminescence emissions, it can be summarized that (1) CH_2_O* as a low-temperature HRR indicator only shows a reliable value at the beginning of the combustion with relatively low intensity. (2) C_2_* and CH* can be used to characterize the flame front and structures. It is shown that both are produced spatially close to the first sharp temperature rise in the reaction zone of a flame. (3) The appearance of the CO_2_* emission appears only after the end of the ignition delay, which is much later than CH_2_O*, CH*, and C_2_*. This is related to CO_2_ is a final production of the combustion. It should be noted that in Fig. 10III and IV the CH_2_O* and CH* signals have been saturated, which might reduce the accuracy of the following analysis.

### Reconstructed chemiluminescence signals

**Figure 11:**
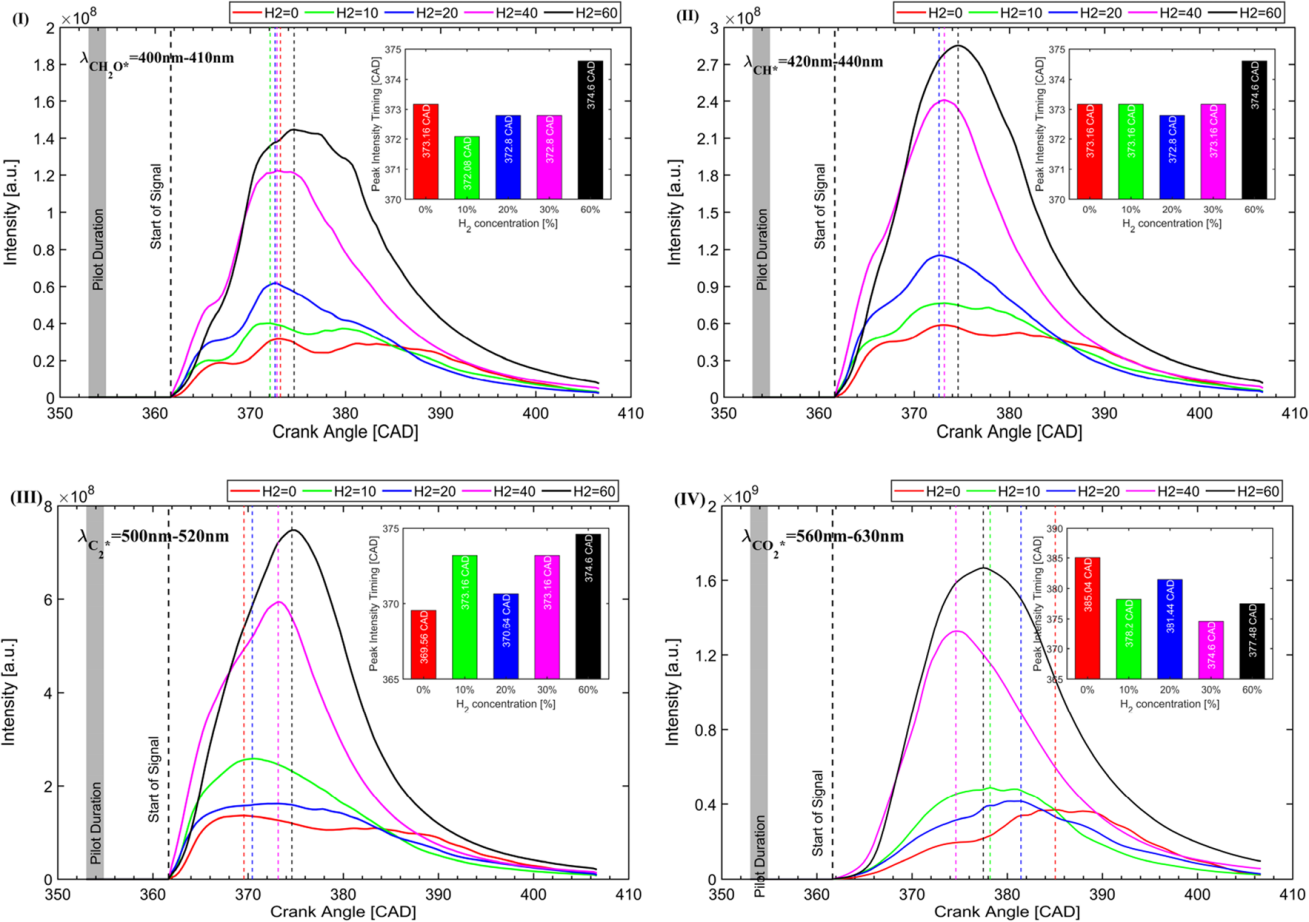
The evolution of the integrated CH_2_O*, CH*, C_2_* and CO_2_* chemiluminescence signal at different ${\chi }_{\text{H}_{2}/\text{CH}_{4}}$ conditions, (**I**) CH_2_O*, (**II**) CH*, (**III**) C_2_*, (**IV**) CO_2_*

Figure 11 summarizes the evolution of the integrated CH_2_O*, CH*, C_2_*, and CO_2_* chemiluminescence signal at different ${\chi }_{\text{H}_{2}/\text{CH}_{4}}$ conditions. As observed in Fig. 10, the addition of H_2_ has an insignificant effect on the intensity of CH_2_O*, CH*, C_2_*, and CO_2_* chemiluminescence when ${\chi }_{\text{H}_{2}/\text{CH}_{4}}\le 40\%$. However, further increasing the H_2_ concentration in the H_2_–CH_4_ mixture leads to a significant increase in the chemiluminescence intensity. It is worth noting that the chemiluminescence intensity of CH_2_O* and CH* monotonically increases with the addition of H_2_, while the chemiluminescence intensity of C_2_* and CO_2_* with ${\chi }_{\text{H}_{2}/\text{CH}_{4}}=10\%$ is higher than that ${\chi }_{\text{H}_{2}/\text{CH}_{4}}=20\%$, then monotonically increases with the addition of H_2_. The interpretation is related to the formation path of these two species. For C_2_* the main formation paths are CH_2_(S) + C = C_2_* + H_2_ and C_3_ + O = CO + C_2_*, which are highly temperature dependent. At low temperatures, the reaction of CH_2_(S) + C = C_2_* + H_2_ is dominant, while increasing the temperature promotes the reaction of C_3_ + O = CO + C_2_*. Since the combustion temperature at ${\chi }_{\text{H}_{2}/\text{CH}_{4}}=20\%$ is not significantly higher than that of ${\chi }_{\text{H}_{2}/\text{CH}_{4}}=10\%$, while there are 10% fewer hydrocarbons involved in the reaction, therefore, a lower chemiluminescence intensity of C_2_* can be observed. Similar to the CO_2_*, the compromisation of the hydrocarbon involved in reactions and combustion temperature results in non-monotonical trends in chemiluminescence intensity.

As explained in the literature^36,37^, the enrichment with H_2_ may influence the CH_2_O*, CH*, C_2_* and CO_2_* chemiluminescence intensities through three major effects: (1) a concentration effect caused by the dilution of the remaining species, thus decreasing the overall light emission; (2) a chemical/kinetic effect caused by the participation of in chemical reactions, which subsequently changes the kinetic pathways through which chemiluminescent species are produced and destroyed; and (3) a thermal-diffusion effect, caused by changes in specific heat capacity and thermal diffusivity in the mixture. The production of CH_2_O*, CH*, C_2_*, and CO_2_* is severely affected by the H_2_ concentration in the charge mixture. The interpretation relies on the addition of the H_2_ promoting the combustion process, which produces more radicals per unit of time. This influences not only the production of these self-excited radicals but also their homonym ground state molecules (CH_2_O, CH, C_2_, and CO_2_), which might also influence chemiluminescent emissions^31^. Additionally, adding the H_2_ also enhances the flame speed and reduces the quenching effects, resulting in more complete combustion subsequent to a larger flame area. This phenomenon can be seen in Figs. 8 and 9 that more view area is captured by the flames. Last but not least, increasing ${\chi }_{\text{H}_{2}/\text{CH}_{4}}$ in the charge mixture also leads to a high combustion temperature in the cylinder, which also affects the chemical kinetics of the reaction and radical emissions. The integrated chemiluminescence signal profile of CH_2_O* and CH* as shown in Fig. 11I and II indicates that the peak intensity of CH_2_O* and CH* takes place at 372.08–374.6 CAD and the addition of the H_2_ has an insignificant effect on the appearance of the peak intensity. However, for C_2_* and CO_2_* as shown in Fig. 11III and IV, increasing the H_2_ fraction in the charge mixture shows a significant effect on the appearance of the emitters. Especially for the CO_2_*, the addition of the H_2_ dramatically advances the CO_2_* emission. Figure 11IV also indicates that the appearance of CO_2_* is much later than the other emitters due to the CO_2_ being the final production.

### 0D simulations

According to the aforementioned NFL analysis, the main emitters of chemiluminescence in hydrocarbon (HC) flames are CH_2_O*, CO_2_*, CH*, OH*, and C_2_* radicals. To further gain an insight into the flame characteristics and combustion chemical kinetics, a 0D simulation in Cantera is elaborated to identify the effect of adding H_2_ into CH_4_/Air mixtures on the formation of electronically excited species. Constant volume 0D homogeneous reactors are considered for this analysis, where H_2_ is added to CH_4_/air, and the peak values of excited species, which take place at the ignition delay time, are reported. Due to the limited availability of chemical kinetics mechanisms that include the formation of excited species, especially for diesel surrogates, two different approaches are considered in this study. These reactions together with the bandhead wavelengths are detailed in Table 3.

**Table 3:** Formation routes (based on elementary reactions) and characteristic wavelengths of excited radicals in a combustion process^38^.

Excited radicals	Formation routes	Transition	Vibrational level	Characteristics wavelength, nm
OH∗	R1:$\mathrm{CH}+{\mathrm{O}}_{2}\to \mathrm{CO}+{\mathrm{OH}}^{*}$	${A}^{2}{\Sigma }^{+}\to {X}^{2}\Pi$	(0,1)	282.9
	R2:$\mathrm{H}+\mathrm{O}+\mathrm{M}\to {\mathrm{OH}}^{*}+\mathrm{M}$		(0,0)	**306.72**
	R3:$\mathrm{OH}+\mathrm{OH}+\mathrm{H}\to {\mathrm{OH}}^{*}+{\mathrm{H}}_{2}\mathrm{O}$		(0,0)	308.9
CH_2_O*	R4:${\mathrm{HO}}_{2}+{\mathrm{CH}}_{2}\to \mathrm{OH}+{{\mathrm{CH}}_{2}\mathrm{O}}^{*}$			**395.2**
	R5:$\mathrm{OH}+{\mathrm{CH}}_{2}\mathrm{O}\to {{\mathrm{CH}}_{2}\mathrm{O}}^{*}+{\mathrm{H}}_{2}\mathrm{O}$			412.0–457.0
	R6: $\mathrm{HCO}+\mathrm{H}+\mathrm{M}\to {{\mathrm{CH}}_{2}\mathrm{O}}^{*}$+M			
CH^∗^	R7:${\mathrm{C}}_{2}+\mathrm{OH}\to \mathrm{CO}+{\mathrm{CH}}^{*}$	${B}^{2}{\sum }_{\mathrm{g}} \to {X}^{2}\prod$	(0,0)	388.9
	R8:${\mathrm{C}}_{2}\mathrm{H}+\mathrm{O}\to \mathrm{CO}+{\mathrm{CH}}^{*}$	${A}^{2}\Delta \to {X}^{2}\prod$	(0,0)	**431.42**
	R9:$\mathrm{H}+{\mathrm{O}}_{2}\to {\mathrm{CO}}_{2}+{\mathrm{CH}}^{*}$			365
C_2_∗	R10:${\mathrm{CH}}_{2}+\mathrm{C}\to {\mathrm{H}}_{2}+{\mathrm{C}}_{2}^{*}$	${A}^{3}{\prod }_{\mathrm{g}}\to {X}^{3}{\prod }_{u}$	(1,2)	471.52
	R11:${\mathrm{C}}_{2}\mathrm{H}+\mathrm{H}\to {\mathrm{H}}_{2}+{\mathrm{C}}_{2}^{*}$		(0,1)	473.71
	R12:${\mathrm{C}}_{3}+\mathrm{O}\to \mathrm{CO}+{\mathrm{C}}_{2}^{*}$		(1,1)	512.93
			**(1,2)**	**516.52**
			(1,0)	563.56
CO_2_∗	R13:$\mathrm{CO}+\mathrm{O}+\mathrm{M}\to \mathrm{M}+{\mathrm{CO}}_{2}^{*}$	${B}^{2}\to {X}^{1}{\Sigma }_{g}^{+}$		**340–650**
	R14:$\mathrm{HCO}+\mathrm{O}\to \mathrm{H}+{\mathrm{CO}}_{2}^{*}$			

Prominent ones are shown in bolded^39^.

The main proposed reaction responsible for OH* formation is mainly formed by reaction (R1) in hydrocarbon flames, whereas its formation in hydrogen flames is due to reactions (R2) and (R3). Unfortunately, the OH* radical emits radiation in the deep-UV range, which can not be detected directly due to the spectral response sensitivity of the color camera starting from 400 nm.

Reactions R4 and R5 present the formation of CH_2_O*, which is characterized by a broadband emission spectrum (350–500 nm). The main formation reactions of CH* can be seen in R7, R8, and R9. The primary CH* emission in the ultraviolet and visible region of the spectrum is due to about 80% of total CH∗ emission stems from the ${A}^{2}\Delta \to {X}^{2}\prod$(∼431 nm) and 20% are from ${B}^{2}{\sum }_{\mathrm{g}} \to {X}^{2}\prod$ (∼390 nm) transitions^36^.

The strongest emission of C_2_* (${A}^{3}{\prod }_{\mathrm{g}}\to {X}^{3}{\prod }_{u}$) can be seen in R10, R11, and R12, named Swan Band, which is responsible for the greenish color with 470–560 nm in flame spectra in stoichiometric and rich hydrocarbon flames^34^.

Unlike the distinct spectroscopic features of OH*, CH_2_O*, CH*, and C_2_*, the chemiluminescence of CO_2_* is characterized by a continuous emission represented by a broadband distributed background signal from 340 to 650 nm, which makes the detailed study of CO_2_* chemiluminescent spectrum difficult^38^.

In the first approach, only CH_4_-H_2_–Air mixtures are considered, and diesel is excluded. The main justification is that the small portion of injected diesel in our experiments has a minor contribution to the flame formation after ignition. Homogeneous CH_4_/H_2_/Air mixtures at ${\chi }_{\text{H}_{2}/\text{CH}_{4}}$ = 0%, 10%, 20%, 40%, 60% with ϕ = 0.5, the pressure of 60 bar, and temperature of 800 K are considered. We note that the selected temperature is arbitrary and by assuming that the charge mixture temperature is increased to 800 K after the diesel ignition and compression effects. The utilized chemical kinetics mechanism is ChemphysMech_v.1^4^ supplemented by reaction blocks of C_2_^*^ and CO_2_^*^ taken along with the coefficients of the thermodynamic polynomials from ref.^40^. In this approach, OH*, CH^*^, C_2_^*^, and CO_2_^*^ are evaluated.

**Figure 12:**
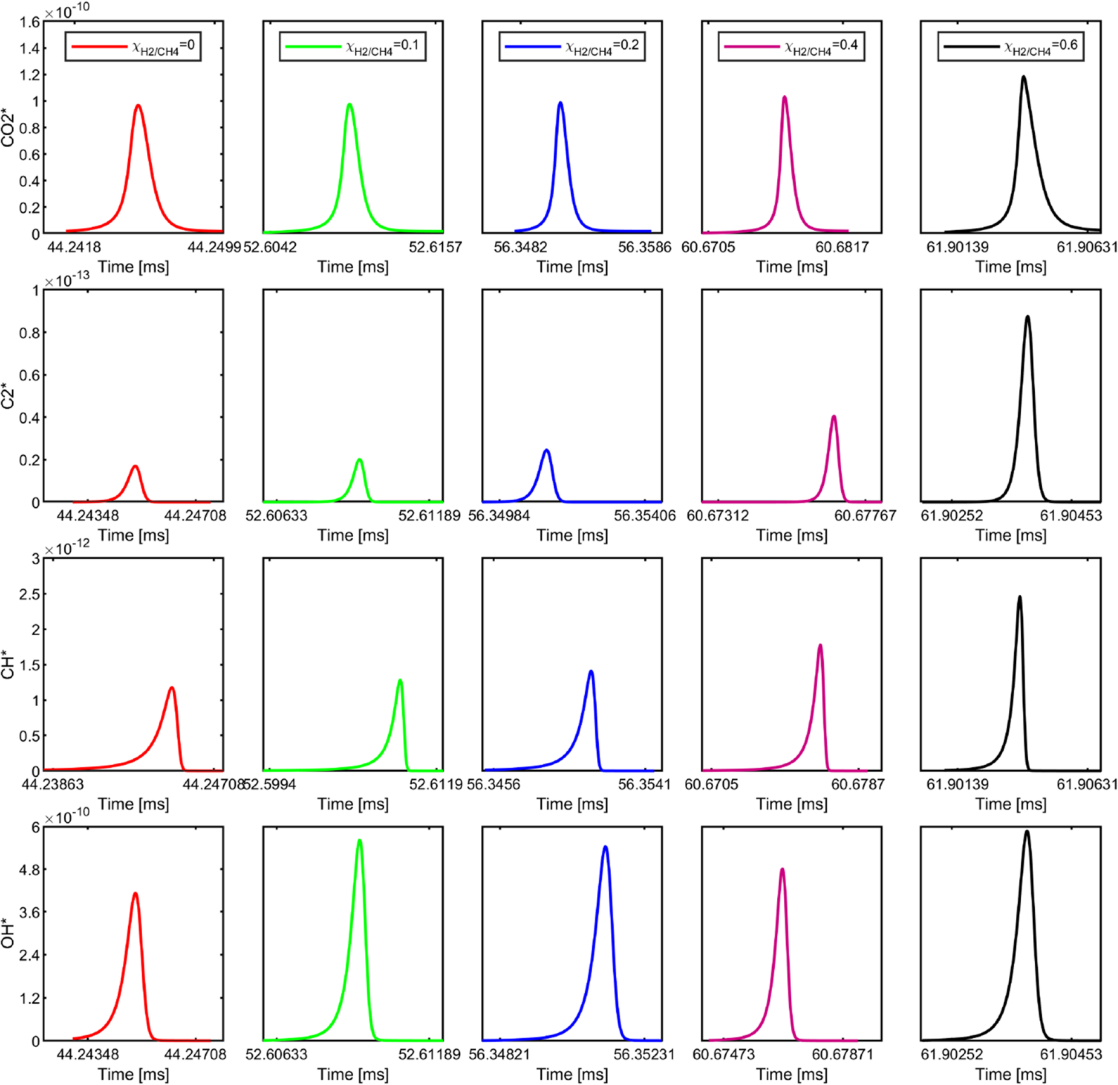
The excited species concentration profiles of CH_4_/H_2_/Air mixture combustion at different ${\chi }_{\text{H}_{2}/\text{CH}_{4}}$.

The excited species profiles for different ${\chi }_{\text{H}_{2}/\text{CH}_{4}}$ are shown in Fig. 12. It is observed that the concentrations of OH* show an insignificant effect on the H_2_ addition. The explanation is that the main formation path of OH* during hydrocarbon combustion is CH + O_2_ = OH* + CO (R1) and a secondary reaction of OH* in hydrocarbon combustion is: H + O + M = OH* + M (R2). Even though the addition of H_2_ increases the reaction R2, however, the lack of the CH* due to the decrease of the hydrocarbons might also slow down the OH* production rate. The chemiluminescence of CH*, C_2_*, and CO_2_* have been used to distinguish rich and stoichiometric reaction zones and HRR indicators in different combustor configurations^39,41–44^. For CH*, A recent shock tube study with CH_4_/H_2_ mixtures supports R7 and R8 as the dominant CH* formation pathways for conditions in the range 1200–2300 K and 0.6–2.2 atm, but R9 could contribute significantly to CH* chemiluminescence in hot flames and under fuel lean conditions. Therefore, when the ${\chi }_{\text{H}_{2}/\text{CH}_{4}}<40\%$, there is no significant effect of H_2_ addition on CH* concentration due to the compensation of the decrease of the hydrocarbon and the flame temperature. However, with further increasing the ${\chi }_{\text{H}_{2}/\text{CH}_{4}}$, the R9 contributes more significantly to the CH* production due to a faster burning rate and higher flame temperature. C_2_* is a prominent emitter in rich CH_4_ flames in the UV–VIS region of the electromagnetic spectrum^34^. Typically, C_2_* bands can be seen for the rich hydrocarbon flames, while for lean hydrocarbon combustion, the C_2_* features are much less pronounced^45^. Therefore, the C_2_* concentration is one order of magnitude less than CH*. A similar trend can be observed with the H_2_ addition on C_2_* and CH*, which is comparable to the experimental results. For CO_2_*, the formation mechanism of CO_2_* has not been fully understood due to its broadband spectra, which is trapping and interfering with the emissions of other species (such as CH_2_O*, CH*, C_2_*, CO_2_*, HCO*, and H_2_O) lead to discrepancies between experiments and simulations. Since the dominance of CO_2_* production is based on R13 at lean conditions, it is less dependent on H_2_ addition. Thus, it can be seen that the addition of H_2_ has an insignificant effect on the CO_2_* concentration.

**Figure 13:**
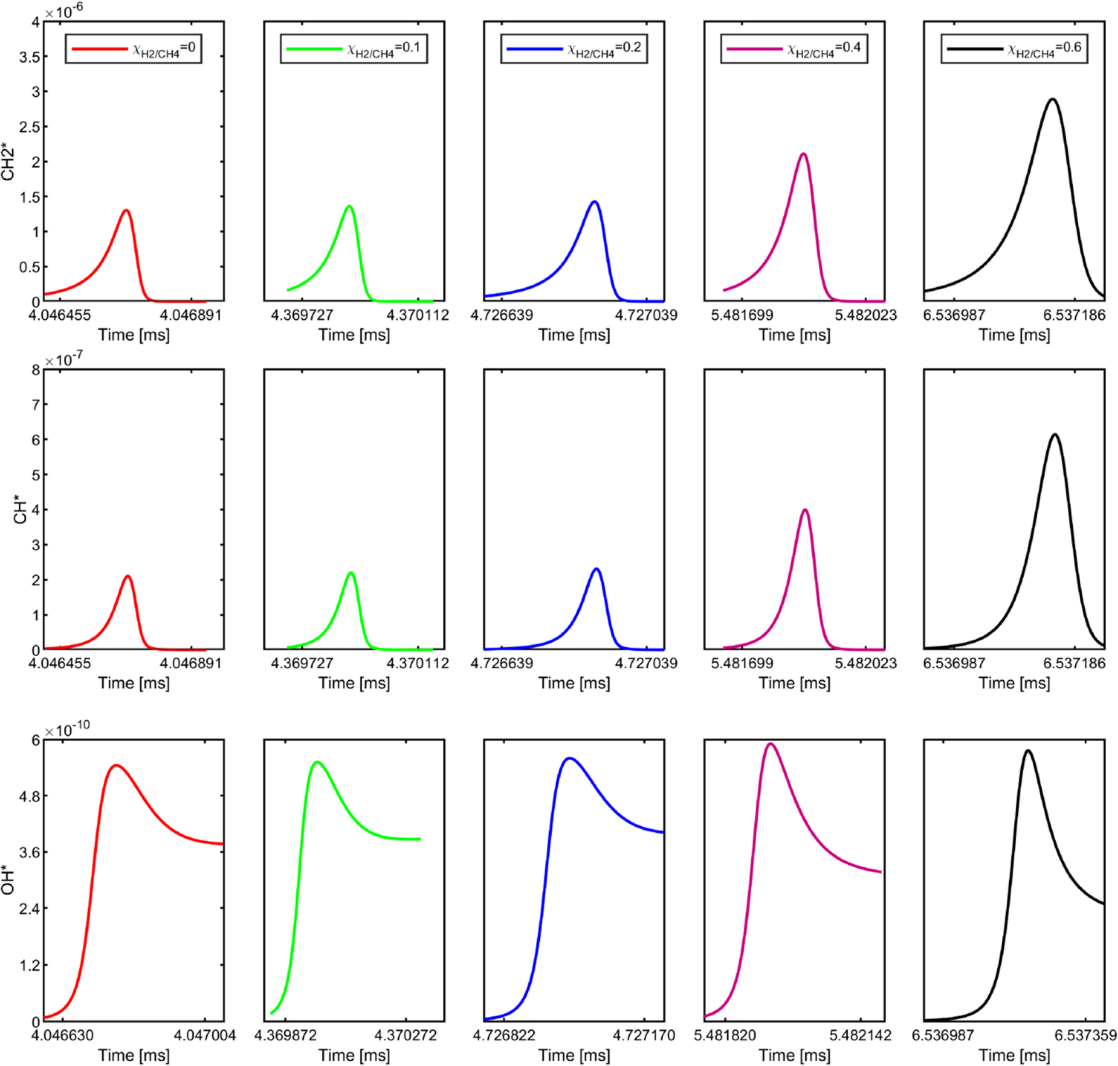
The excited species concentration profiles of n-dodecane/CH_4_/H_2_/Air mixture combustion at different ${\chi }_{\text{H}_{2}/\text{CH}_{4}}$.

In the second approach, the mixing line concept is described by Karimkashi et al.^46^. Here, n-dodecane is considered the diesel surrogate for the cold stream at 363 K, while CH_4_/H_2_ mixtures at ϕ = 0.5 are the hot stream at 800 K. For each mixture of methane/hydrogen (${\chi }_{\text{H}_{2}/\text{CH}_{4}}$) as the hot stream and n-dodecane as the cold stream, the most reactive mixture fraction is found, and the 0D homogeneous reactor simulation results are reported. The most reactive mixture fractions for ${\chi }_{\text{H}_{2}/\text{CH}_{4}}$ = 0%, 10%, 20%, 40%, and 60% are Z^mr^ = 3.5*10^–2^, 3.5*10^–2^, 3.5*10^–2^, 4.0*10^–2^, 4.5*10^–2^, and T^MR^ = 766.7, 766.7, 766.7, 762.8, and 758.9 K, respectively. In this approach, the chemical kinetics mechanism by Banerjee et al.^47^ is considered. In this approach, only OH*, CH^*^, and CH_2_* are evaluated due to the lack of the kinetic mechanism of other chemiluminescence species under such conditions. Thus, only CH* can be used for experimental and simulation results comparison. Similar to the observations for CH_4_/H_2_/Air, increasing ${\chi }_{\text{H}_{2}/\text{CH}_{4}}$ leads to higher concentrations of CH*, as shown in Fig. 13. It should note that the presence of the more reactive fuel n-dodecane, significantly reduces the ignition delay time. Moreover, since CH_2_ is the main reactant for the CH_2_O* and C_2_*, the effect of the H_2_ concentration on the CH_2_* emission could provide an approximation of the CH_2_O* and C_2_* prediction.

## Conclusion

Natural flame luminosity (NFL) imaging is adopted to acquire the flame features and combustion characteristics in an optical engine by a high-speed color camera. The spectral footprint and evolution of the flames in the optical engine are investigated using a physically plausible spectral reconstruction approach to reconstruct the hyperspectral images from RGB NFL images. The TF combustion using premixed CH_4_/H_2_ mixtures ignited by a diesel pilot is comprehensively investigated with varying H_2_ fractions (${\chi }_{\text{H}_{2}/\text{CH}_{4}}$  = 0%, 10%, 20%, 40%, 60%) is examined by NFL imaging and spectral reconstruction. To theoretically gain an insight into the chemiluminescence mechanism and combustion kinetics, a detailed kinetic mechanism for chemiluminescence species such as OH*, CH*, C_2_*, and CO_2_* is adopted to describe the spectral emissions of H_2_/CH_4_ and H_2_/CH_4_/n-dodecane combustion at relevant conditions. According to the analysis of experimental and numerical data, the following conclusions and implications for the evaluation of natural-luminosity images can be stated:

1. Adding H_2_ into the CH_4_/air mixture promotes the flame speed and burning rate, which leads to brighter flame luminosity and a larger engulfing flame area in the cylinder due to the broader flammability limit and higher flame speed of the H_2_ than CH_4_.
2. A PPSR algorithm is successfully implemented for hyperspectral image reconstruction from true-color NFL images. There are two spectral peaks (570 nm and 680 nm), which represent the strongest spectral emissions during the combustion. The addition of the H_2_ mostly broadens the peak spectral at a shorter wavelength and narrows the longer wavelength.
3. Four chemiluminescent radicals (CH_2_O*, CH*, C_2_*, CO_2_*) in the visible range are selected to characterize the flame evolution and chemical reaction during the combustion. The CH_2_O*, CH*, and C_2_* appears at the same time after ignition, which is earlier than that of CO_2_*. The CH_2_O* shows the lowest intensity due to the decomposition and oxidation that might take place at high temperatures, while CO_2_* exhibits the highest intensity due to its border broadband and high concentration at the end of combustion.
4. Detailed 0D chemical kinetics simulations are conducted with CH_4_/H_2_/Air and n-dodecane/ CH_4_/H_2_/Air at engine-like conditions to evaluate the effect of H_2_ fraction on electronically excited reactive species. The results indicate that the addition of H_2_ has an insignificant effect on the CO_2_* emissions, however, the concentration of CH* and C_2_* increases with the addition of H_2_, especially when ${\chi }_{\text{H}_{2}/\text{CH}_{4}}\ge 40\%$. The numerical results show a similar tendency to the experimental results.

In summary, the novelty of the work consists of the following three features: (1) true-color NFL imaging is adopted to identify the flame characteristics in an optical engine under ultra-lean CH_4_/H_2_/Diesel TF combustion mode, (2) a physically plausible spectral reconstruction algorithm is applied to reconstruct the spectral information from RGB images, (3) four key chemiluminescent radicals are selected to identify the effect of H_2_ concentration on chemical kinetics coupled with 0D chemical kinetics simulation.

## Data Availability

The datasets used and/or analyzed during the current study are available from the corresponding author on reasonable request.
